# Genome-Wide Identification of Binding Sites Defines Distinct Functions for *Caenorhabditis elegans* PHA-4/FOXA in Development and Environmental Response

**DOI:** 10.1371/journal.pgen.1000848

**Published:** 2010-02-19

**Authors:** Mei Zhong, Wei Niu, Zhi John Lu, Mihail Sarov, John I. Murray, Judith Janette, Debasish Raha, Karyn L. Sheaffer, Hugo Y. K. Lam, Elicia Preston, Cindie Slightham, LaDeana W. Hillier, Trisha Brock, Ashish Agarwal, Raymond Auerbach, Anthony A. Hyman, Mark Gerstein, Susan E. Mango, Stuart K. Kim, Robert H. Waterston, Valerie Reinke, Michael Snyder

**Affiliations:** 1 Department of Molecular Cellular Developmental Biology, Yale University, New Haven, Connecticut, United States of America; 2 Department of Molecular Biochemistry and Biophysics, Yale University, New Haven, Connecticut, United States of America; 3 Max Planck Institute for Molecular Cell Biology and Genetics, Dresden, Germany; 4 Department of Genome Sciences, School of Medicine, University of Washington, Seattle, Washington, United States of America; 5 Department of Genetics, Yale University, New Haven, Connecticut, United States of America; 6 Huntsman Cancer Institute, University of Utah, Salt Lake City, Utah, United States of America; 7 Program in Computational Biology and Bioinformatics, Yale University, New Haven, Connecticut, United States of America; 8 Departments of Developmental Biology and Genetics, Stanford University Medical Center, Stanford, United States of America; 9 Department of Molecular and Cellular Biology, Harvard University, Cambridge, Massachusetts, United States of America; The University of North Carolina at Chapel Hill, United States of America

## Abstract

Transcription factors are key components of regulatory networks that control development, as well as the response to environmental stimuli. We have established an experimental pipeline in *Caenorhabditis elegans* that permits global identification of the binding sites for transcription factors using chromatin immunoprecipitation and deep sequencing. We describe and validate this strategy, and apply it to the transcription factor PHA-4, which plays critical roles in organ development and other cellular processes. We identified thousands of binding sites for PHA-4 during formation of the embryonic pharynx, and also found a role for this factor during the starvation response. Many binding sites were found to shift dramatically between embryos and starved larvae, from developmentally regulated genes to genes involved in metabolism. These results indicate distinct roles for this regulator in two different biological processes and demonstrate the versatility of transcription factors in mediating diverse biological roles.

## Author Summary

The *C. elegans* transcription factor PHA-4 is a member of the highly conserved FOXA family of transcription factors. These factors act as master regulators of organ development by controlling how genes are turned off and on as tissues are formed. Additionally they regulate genes in response to nutrient levels and control both longevity and survival of the organism. However, the extent to which these factors control similar or distinct gene targets for each of these functions is unknown. For this reason, we have used the technique of chromatin immunoprecipitation followed by deep sequencing (ChIP–Seq), to define the target binding sites of PHA-4 on a genome-wide scale, when it is either functioning as an organ identity regulator or in response to environmental stress. Our data clearly demonstrate distinct sets of biologically relevant target genes for the transcription factor PHA-4 under these two different conditions. Not only have we defined PHA-4 targets, but we established an experimental ChIP–Seq pipeline to facilitate the identification of binding sites for many transcription factors in the future.

## Introduction

A major scientific endeavor is aimed toward understanding how the regulatory information embedded in the genome is deployed to direct the complex process of development [1]. With the completion of the genomic sequence of many model organisms, much effort is now focused on identifying the precise regions of the genome that regulate specific developmental events. Of particular interest are regions that serve as binding sites for developmentally important transcription factors. Through these sites, a transcription factor controls the spatial and temporal expression of genes that function in diverse developmental processes. Identification of the DNA binding sites of a factor links it to its direct target genes, and permits a fuller understanding of the mechanisms by which different transcription factors control the development of an organism.

Ultimately, understanding transcriptional regulation of development requires identification of the regulatory network as a whole. The binding sites of many transcription factors under similar conditions must be determined, as well as how binding sites for a given transcription factor change over time as development progresses. To this end, we have developed a high-throughput experimental system to categorize the binding sites of many transcription factors using chromatin immunoprecipitation (ChIP) in the developmental model organism, the nematode *C. elegans*.

*C. elegans* provides many advantages to deciphering developmental regulatory networks [2]. The invariant cell lineage of *C. elegans* provides an excellent framework to interpret how regulatory networks control development. Additionally, the spatial and temporal expression of both transcription factors and their targets can be followed using live GFP imaging techniques. The small size and simple growth conditions of *C. elegans* facilitate large-scale biochemical analyses such as ChIP. Finally, because the genome is relatively compact, individual genes are small and close together, which simplifies multiple steps of the process, from cloning procedures to downstream bioinformatics analysis.

We have established an experimental system to systematically tag *C. elegans* transcription factor genes with a fluorescent epitope tag, create transgenic animals expressing a tagged factor, and perform chromatin immunoprecipitation followed by deep sequencing (ChIP-Seq) to identify binding sites for that factor [3],[4]. We first applied this approach to the large subunit of RNA polymerase II, AMA-1, and demonstrate that tagged AMA-1 can recapitulate binding by endogenous AMA-1. We then focused on the sequence-specific transcription factor PHA-4/FOXA because of its well-studied role as a master regulator of pharynx development during embryogenesis [5]–[7], as well as a novel role in improved survival under starvation conditions that we describe here. We identified binding sites for PHA-4/FOXA under two developmental conditions: during embryogenesis and during the first stage of larval development (L1) under starvation conditions. We found that the binding sites and associated gene targets of PHA-4 in embryogenesis are generally associated with organ development, whereas the targets in L1 are primarily associated with metabolism, reflecting the expected biology of each condition. Interestingly, we find that several targets preferentially bound in starved L1s are involved in autophagy. These data establish that we have laid the foundation for systematic identification of genome-wide transcription factor binding sites during *C. elegans* development and demonstrate new roles for key regulators in diverse biological processes.

## Results

### Establishing a pipeline for systematic analysis of transcription factor binding sites

**Figure 1:**
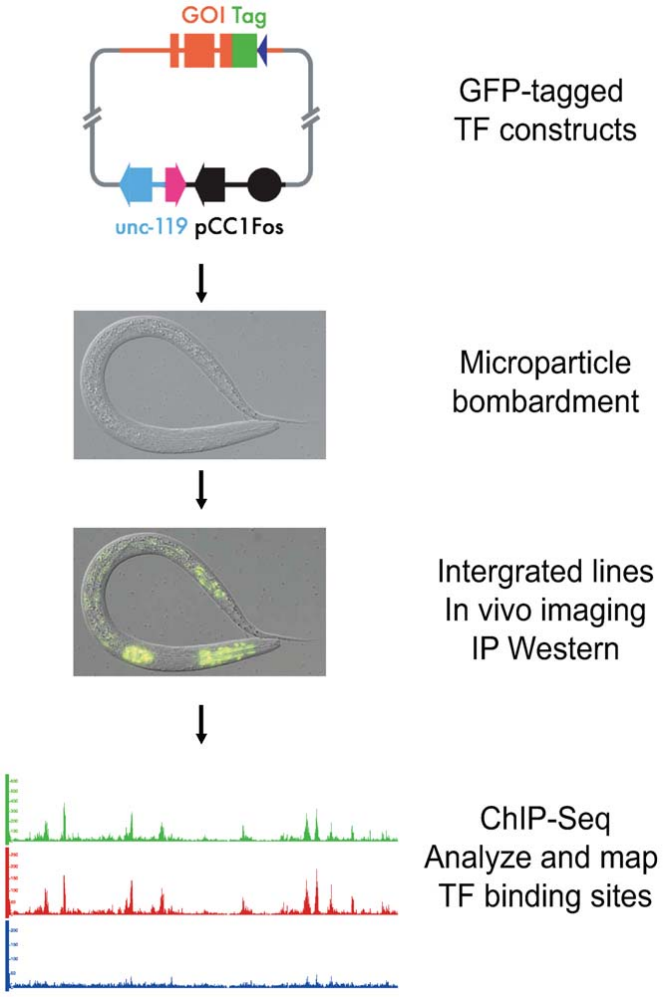
Experimental pipeline for identification of transcription factor binding sites in *C. elegans*. Individual transcription factors encoded within fosmids are tagged with a dual green fluorescent protein (GFP) and 3xFLAG tag at its C-terminus. A construct is then bombarded into worms to generate a series of integrated transgenic lines expressing the tagged factor. The expression of each transcription factor is confirmed through both fluorescence imaging and immunoblot analysis. The binding sites of each transcription factor are then identified using chromatin immunoprecipitation followed by high-throughput sequencing (ChIP-Seq).

Identification of binding sites in vivo is critical to understand how transcription factors operate in regulatory networks to control development. We therefore have established a pipeline to facilitate this endeavor in *C. elegans* (Figure 1). To briefly summarize, we first generated constructs in which each transcription factor is tagged in frame with a dual GFP:3xFLAG tag at the carboxyl terminus. This tag provides both direct visual evidence of spatial and temporal expression in vivo, as well as two different epitopes that can be utilized for biochemical experiments. We used recombineering to insert the tag directly into a fosmid that contains the entire locus of interest as well as extensive flanking regions (Sarov et al., in prep). This approach increases the likelihood that the transcription factor will have the essential regulatory information to allow it to be expressed correctly in vivo.

These constructs were introduced into worms via microparticle bombardment, which produces animals bearing low-copy number, integrated transgenes [8]. We then isolated lines carrying an integrated transgene, and examined them for expression of the GFP-tagged factor by fluorescence microscopy. We determined the developmental stage at which maximal expression occurs, as well as whether the transcription factor is localized to the nucleus as expected. Additionally, we examined the size of the tagged transgenic protein by immunoblot analysis using both anti-FLAG and anti-GFP antibodies. Finally, we tested whether the protein can be immunoprecipitated with an antibody to GFP (anti-GFP; Materials and Methods), followed by immunoblot analysis.

If all of these quality control measures were passed, we then grew the transgenic animals to the desired developmental time, harvested and crosslinked the sample, and performed chromatin immunoprecipitation (ChIP) using anti-GFP to collect chromatin preferentially bound by the GFP-tagged factor [9]. This chromatin was then subjected to Illumina-based sequencing, as was non-immunoprecipitated (input) chromatin from the same sample, which served as a control.

### GFP-tagged AMA-1 has the same binding pattern as native AMA-1

We examined whether a transgenic tagged factor could recapitulate the binding sites of the endogenous, untagged factor. To directly compare the binding properties of a tagged factor expressed from a transgene with that of the endogenous protein, we first performed ChIP for both the tagged and native versions of AMA-1, the large subunit of RNA polymerase II (RNA Pol II). AMA-1 is well-suited for this comparison because commercially available antibodies against RNA Pol II recognize *C. elegans* AMA-1, and perform well in ChIP assays [10],[11]. Additionally, AMA-1 is abundantly expressed in the nucleus of all cells of the animal [12],[13].

**Figure 2:**
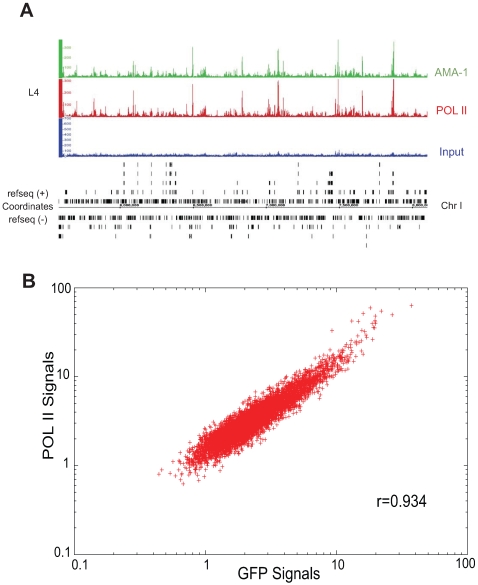
Binding patterns of GFP-tagged AMA-1 are highly similar to that of native AMA-1. (A) Signal tracks of AMA-1 binding profiles for a representative stretch of chromosome I. The top track represents binding of AMA-1:GFP as detected by anti-GFP. The middle track represents binding of AMA-1 and AMA-1:GFP as detected by anti-RNA Pol II (8WG16). The bottom track represents input chromatin. (B) Signal values (relative abundance of sequencing tags in ChIP DNA versus input) for each binding site (p<0.001) in the anti-GFP and anti-8WG16 IPs were subjected to Pearson correlation coefficient analysis across 600 bp windows. The linear correlation coefficient between the two samples is 0.934.

We therefore established a transgenic strain that expresses AMA-1:GFP:3xFLAG (referred to as AMA-1:GFP thereafter) in all nuclei, recapitulating the wild type expression pattern (Figure S1A). We grew duplicate populations of AMA-1:GFP animals to the L4 stage, which was chosen to provide a stringent test case, as it provides a particularly biologically complex stage that can be difficult to replicate. We then performed ChIP using two different antibodies: anti-GFP, which recognizes the tagged AMA-1, and anti-Pol II (8WG16, pan-Pol II), which recognizes both tagged and native proteins in both phosphorylated and non-phosphorylated forms (Figure S1B). The DNA from each immunoprecipitation was purified and the ends subjected to sequencing using the Illumina platform, as was input DNA isolated from crosslinked and sonicated cells (non-immunoprecipitated). The binding profiles of both samples were determined and then compared (Figure 2A; Figure S1C). The overall correlation between anti-GFP and anti-Pol II ChIP samples was extremely high (0.934; Figure 2B), indicating that the tagged AMA-1:GFP had a binding profile highly similar to that of native RNA Pol II. Indeed, the correlation between IPs performed on the same biological samples was higher than that for IPs performed with the same antibody on different biological replicates (Figure S1C). Importantly, the tagged AMA-1 did not exhibit significant ectopic binding sites not found with endogenous RNA Pol II, indicating that the transgenic system does not induce a major increase of non-specific binding. Moreover, the binding sites identified in the two samples have the similar characteristic of broad peaks distributed over the length of the gene, as expected for genes undergoing active transcription (Figure S2). We conclude that the addition of the GFP:3xFLAG tag does not disrupt the ability of AMA-1 to interact with its endogenous target genes, and that our anti-GFP antibody works very well in ChIP experiments in *C. elegans*.

### PHA-4 chromatin immunoprecipitation identifies thousands of binding sites

We next determined the binding sites for a key transcription factor, PHA-4/FOXA. PHA-4 is a master organ identity gene that is required for the specification and formation of the pharynx. The expression of several hundred genes in the developing embryonic pharynx is dependent upon PHA-4, many of which are likely direct targets [14],[15]. Moreover, PHA-4 is required continuously after birth [14] and plays a role in diet-induced longevity in adults, in the absence of another FOX family transcription factor, DAF-16 [16],[17].

**Figure 3:**
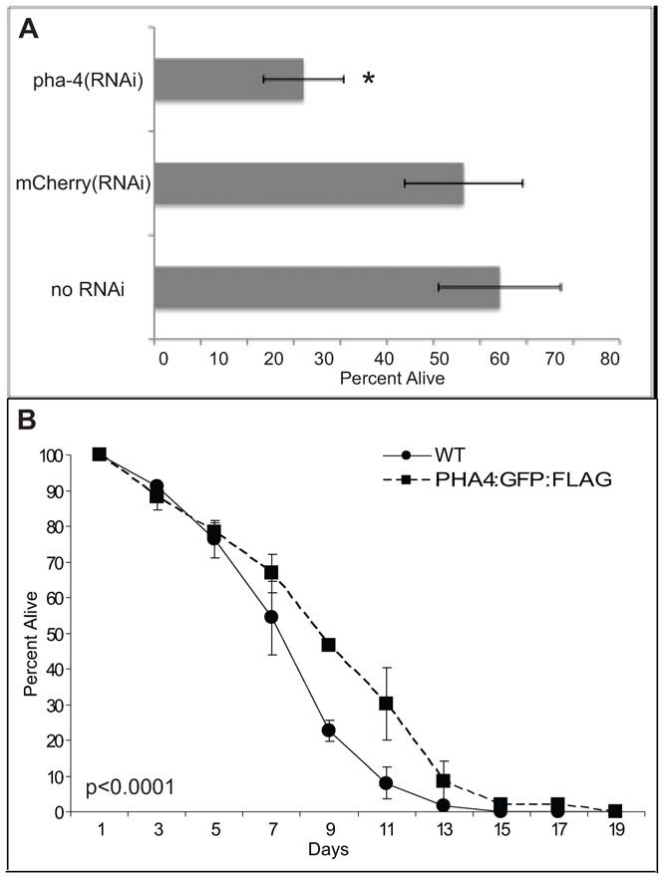
PHA-4 is required for starvation survival. (A) Loss of *pha-4* leads to reduced starvation survival of first stage (L1) larvae. Wild-type (WT) worms were soaked in no RNAi, Cherry double-stranded RNA (dsRNA), or *pha-4* dsRNA without food for the indicated times. To determine viability, triplicate samples were transferred to plates with food. Numbers of worms surviving past L1 were counted after 2 days. Results are an average of three independent experiments, n = 300–500 worms counted for each strain per experiment, error bars represent standard error. * = p<0.05. (B) Overexpression of *pha-4* increases L1 starvation survival. PHA-4:GFP and outcrossed WT worms were subjected to starvation in liquid. Survival was determined as in (A). Results are an average of two independent experiments, n = 500–1900 worms counted for each strain per experiment, error bars represent standard error, p<0.0001 log rank (Mantel-Cox) test.

In addition to these previously described functions, we discovered an additional function for PHA-4 in promoting the survival of first stage larvae (L1) undergoing starvation (Figure 3). L1 animals were transiently subjected to *pha-4(RNAi)* or a negative control Cherry(RNAi) and incubated in the absence of food (Materials and Methods). After eight days of starvation, larvae were transferred to food and tested for their ability to mature beyond the L1 stage. *pha-4(RNAi)* animals exhibited a significantly reduced survival rate at 30%, compared to 75% from the negative control (Figure 3A). However, no difference in survival was observed for up to four days of starvation, indicating that *pha-4(RNAi)* larvae were healthy, and had not suffered developmental defects (data not shown). Conversely, transgenic expression of *pha-4* from its native promoter was sufficient to prolong starvation survival relative to a control, from a mean survival of 8.3±0.2 days in wild type to 9.4±0.2 days in a strain expressing tagged PHA-4 (Figure 3B). Thus, PHA-4 participates in diverse biological processes at different stages of development, with roles in embryonic pharynx development, L1 starvation survival, and adult longevity.

**Figure 4:**
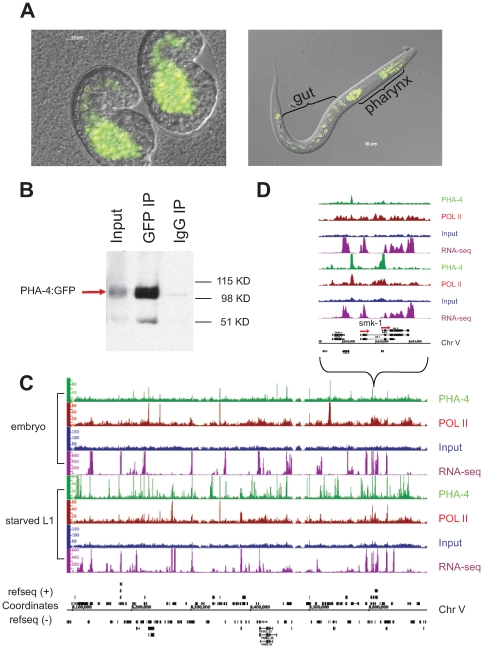
Identification of PHA-4 binding sites in embryos and starved L1 larvae. (A) PHA-4:GFP is expressed primarily in the pharynx and gut in embryos and L1s. (B) PHA-4:GFP is enriched upon immunoprecipitation by anti-GFP relative to input and is not immunoprecipitated by a control IgG antibody. (C) Signal tracks demonstrating specific PHA-4:GFP binding sites on chromosome V. Green track – PHA-4:GFP (GFP antibody); maroon track – RNA Pol II (8WG16 antibody); blue track – input control; purple track – mapped reads from RNA sequencing data. Embryonic data set shown on top, L1 larval dataset shown below. (D) Close-up of *smk-1* locus showing that PHA-4 binding changes between stages, although the gene appears to be expressed at both stages. Other examples of PHA-4 binding are shown in Figure S5.

To identify PHA-4 binding sites in the genome, PHA-4:GFP:3xFLAG (referred to as PHA-4:GFP thereafter) transgenic animals were created via our pipeline. Animals bearing an integrated transgene had nuclear-localized expression in the pharynx and intestine in embryos, and in pharynx, intestine and rectum in larvae, confirming published expression patterns [5]–[7],[18] (Figure 4A). Moreover, immunoblot analysis using anti-GFP demonstrated a tagged protein somewhat larger than 90kD, the approximate size expected of the largest PHA-4 isoform containing the GFP:3xFLAG tag (Figure 4B). Finally, we crossed animals bearing the PHA-4:GFP transgene to *pha-4(q90)* mutants, and rescued the embryonic lethality of these mutants.

To compare the binding patterns of PHA-4 under different conditions, we collected and crosslinked PHA-4:GFP transgenic animals during embryogenesis when the pharynx is forming, and during the L1 larval stage under starvation conditions. Biologically independent duplicate samples were collected. The samples were immunoprecipitated with anti-GFP to identify PHA-4 binding sites, and also with anti-RNA Pol II antibodies to identify the location of RNA Pol II, which will help to define the transcriptional state of genes associated with PHA-4. The immunoprecipitated chromatin, along with control input DNA from the same animals, was then sequenced to the depth of >10^6^ reads per sample. Figure 4C shows the binding patterns of PHA-4 and RNA Pol II at both developmental times for a representative region of the genome, as well as a closer view of the binding patterns at the gene *smk-1*. *smk-1* encodes a potential co-factor for PHA-4 [16]; our data suggest that it might also be a regulatory target of PHA-4 (Figure 4D). We also collected RNA from wild type embryos and L1 samples and performed cDNA deep sequencing [19] to identify expressed genes through an independent method.

**Figure 5:**
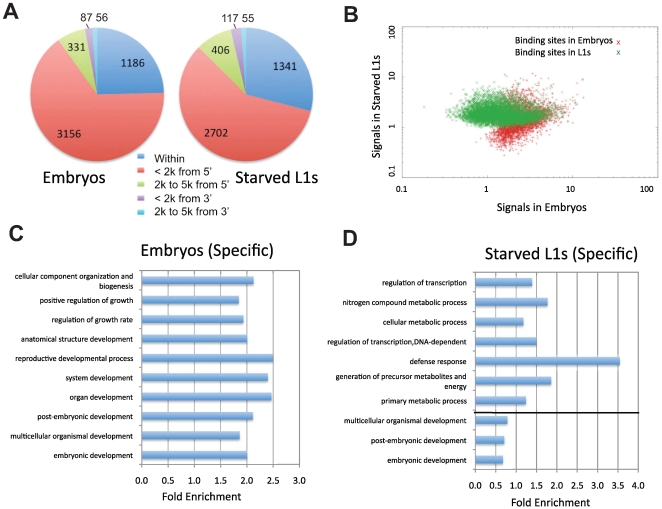
Characterization of PHA-4 binding patterns and gene targets. (A) The distribution of the distance between PHA-4 binding sites and candidate gene targets (Figure S3 for algorithm for assigning gene targets). (B) Scatter plot comparing similarity and uniqueness of PHA-4 binding profile in embryos and L1 larvae. Signal strength is sequenced reads normalized against input in the peak region (p<10^−5^). (C,D) Gene Ontology (GO) categories showing the highest level of enrichment for the candidate target genes of PHA-4 specific to embryos (C) and L1 larvae (D). Fold enrichment is defined as the increase in abundance in the immunoprecipitated sample relative to total input chromatin.

Using a peak-scoring algorithm [20], we identified discrete PHA-4 binding sites for each sample. A total of 4350 and 4808 binding sites were defined in embryos and starved L1 larvae, respectively (p<10^−5^; Table 1 and Dataset S1, S2). We found a high correlation between replicate experiments for each stage (0.85 for embryos, 0.88 for L1; data not shown). We also developed a target-calling algorithm (Figure S3) to assign these binding sites to candidate gene targets. Genes within 2 kb of a binding site were designated as candidate regulatory targets of PHA-4, which resulted in the assignment of over 90% of the sites to one or more genes (Figure 5A). A binding site could be assigned to more than one gene if it fell within a gene-dense interval (Materials and Methods). In total, 4816 protein-coding genes are candidate PHA-4 targets in embryos, and 4621 genes are candidate PHA-4 targets in L1 larvae. Only 280 binding sites lie >5 kb from annotated genes. Presumably these either act a distance or regulate genes that have not yet been annotated, such as non-protein coding genes. Overall, these data indicate that PHA-4 has a broad role in directly regulating the expression of many genes in the *C. elegans* genome, in agreement with previously published studies [14].

**Table 1:** Summary of PHA-4 binding sites and gene targets.

	Total binding sites	Total targeted genes^1^	Targeted genes for GO Analysis^1^^,^ 2	Unique targets
**embryos**	4350	4816	4342 (2487)^3^	1975 (1328)
**Starved L1s**	4808	4612	4043 (2062)	1676 (905)

1p value is less than 1×10^−5^.

2The binding site is within the gene or less than 2kb to the 5′ end of gene.

3The value in the parentheses is the number of genes annotated by GO.

We used several methods to validate these binding sites. We first used ChIP-qPCR to directly test whether we could detect enriched binding of PHA-4 at 94 individual candidate sites taken from both embryonic and L1 data sets (Table S1). We found that 76% of the embryonic sites and 74% of L1 sites were reproducibly enriched two-fold or higher by ChIP-qPCR of a biologically independent replicate. Thus, many PHA-4 binding sites identified by ChIP-Seq are verified through an independent detection method. Additionally, we compared our results to an earlier expression analysis that had identified genes expressed during pharynx development in embryos [14] (Figure S4, Materials and Methods). We compared our list of genes to the list of known of pharynx development genes and found that over 38% were bound by PHA-4 in our embryonic ChIP-Seq experiment, which is significantly higher compared to a randomized set (90/238; p<1.7×10^−13^). Moreover, seven of these pharynx-expressed genes had been previously demonstrated to be bound directly by PHA-4 using a gel shift assay [14], and six of the seven were bound by PHA-4 in our experiments at sites containing the previously identified PHA-4 consensus sequence.

Finally, we examined the sequence underlying PHA-4 binding peaks to identify de novo consensus binding sequences enriched under the peaks relative to the genome. Five of the six consensus sequences identified in either of the two stages were variations of the known PHA-4 binding consensus sequence TRTTKRY [14], primarily TGTBTSY (B = [TGC], S = [GC], Y = [TC], p<10^−4^) (Figure S5). Intriguingly, the PHA-4 binding site sequence in embryos differs from those identified in starved L1s. Moreover, a second, unrelated site was identified in embryos that was not found in starved L1s, GAGAGAG/C (3.3-fold; p<10^−26^). This GAGA element, was previously noted as associated with timing of pharynx development in embryos [15]. The GAGA sequence was not enriched among PHA-4 binding sites in starved L1 larvae, or in a control dataset consisting of HTZ-1 binding peaks [11], indicating that it is specific to the PHA-4 embryo dataset. These observations suggest that PHA-4 might have different co-factors at the two developmental stages that direct it to distinct targets and distinct binding sites in response to developmental and environmental cues. We conclude that many of the global PHA-4 binding sites we have identified likely reflect functionally relevant binding events in the *C. elegans* genome.

### PHA-4 binding profiles are developmentally regulated

To determine the degree to which binding sites change under different conditions, we compared the PHA-4 binding profiles at the two stages (Figure 5B). The two datasets exhibit extensive overlap (2367 targets), but also have many sites present in one stage but not the other. Of the PHA-4 embryogenesis targets, 1975 (45%) are not found on the list of PHA-4 L1 targets, while 1676 (41%) of PHA-4 L1 targets are not found among the embryo set. This observation indicates that the binding profile of PHA-4 shifts substantially under distinct developmental conditions.

To globally categorize the types of genes that are differentially regulated, we determined the Gene Ontology (GO) functional categories that are enriched among each set of stage-specific PHA-4 targets (Figure 5C and 5D). We found that the embryo set is enriched for developmental processes, whereas the L1 set of targets is enriched for metabolic processes and defense responses (Table S2 and Table S3). Although these functional categories are quite broad, this shift in the basic functions of the targets is consistent with the shift in the function of PHA-4 from organ development to an altered metabolic response to promote survival of starvation conditions.

To investigate these differences in greater detail, we individually annotated a subset of candidate gene targets. We first selected target genes based on the presence of strong binding sites (p<10^−10^) 0–2kb upstream of the gene, in the candidate regulatory region. The subset of those targets that had already been assigned a three-letter name, and presumably had some functional information available, were then divided into common, embryo-only, and starved L1-only sets consisting of 202, 312, and 294 genes, respectively (Dataset S3). We explored gene function by reviewing available data summaries in public databases, such as Wormbase, and noting multiple trends and distinctions between the datasets (Table S4). Many genes throughout all three datasets have been described as expressed in pharynx or intestine, or are known to have a role in muscle development or function. Additionally, genes encoding ribosomal proteins are targeted in all three datasets, with the most found in the common, or shared, dataset. Intriguingly, multiple components of the RNAi pathway are also candidate PHA-4 targets, as are splicing regulatory factors.

Several striking differences were obvious between the two developmental conditions we examined. For instance, the target set in embryos includes many components of G-protein signaling, but the L1 set was devoid of this signaling pathway. Conversely, the L1 set had multiple examples of modulators of the TGFβ-signaling pathway, which is involved in controlling both body size and dauer formation [21], whereas the embryo set did not. Additionally, the embryo set contains many genes that encode chromatin regulators, including multiple members of the SynMuv B pathway, NuRD components, and histone modifying proteins. Intriguingly, multiple members of the dosage compensation machinery are apparently targeted by PHA-4 binding in embryos, such as *dpy-22*, *dpy-27*, *dpy-30*, and *sdc-2*. In contrast to the embryo set, the L1 set of targets with likely roles in transcription primarily consist of sequence-specific transcription factors rather than chromatin-modifying proteins. Most notably, over five times as many nuclear hormone receptors were bound by PHA-4 in starved L1s compared to embryos (28 vs. 5, respectively). Additionally, the metabolism-related factors in starved L1s consist largely of multiple regulators of sterol and fatty-acid metabolism, as well as cytochrome P450 and glutathione-S-transferase components. The starved L1 set also include several components involved in acetylcholine metabolism and signaling, which is involved in neuromuscular synapse transmission. Starved L1s also have increased PHA-4 binding at various types of membrane-bound proteins, including several multidrug resistance proteins, P-glycoproteins, tetraspanins, and serpentine receptors. Many fewer of these types of proteins were noted in the shared or embryo sets.

This shift in functions between stages is exemplified by PHA-4 target genes involved in autophagy. Autophagy in multicellular organisms can be induced by environmental stresses including food limitation. Moreover, autophagy genes are essential for dauer development and life-span extension by diet restriction in *C. elegans* [22]–[24]. Recent genetic assays indicate that the autophagic response to dietary restriction is a transcriptionally regulated response that requires PHA-4 activity [24]. Four genes known to be involved in autophagy (*bec-1*, *lgg-1*, *gpd-2*, and *unc-51*) and found that all four are strongly bound by PHA-4 in starved L1 larvae, but PHA-4 exhibits minimal binding in embryos (Figure S6). Thus, our data suggest that PHA-4 is directly involved in inducing the expression of autophagy genes in response to starvation.

### PHA-4 preferentially associates with transcriptionally poised or active genes

**Figure 6:**
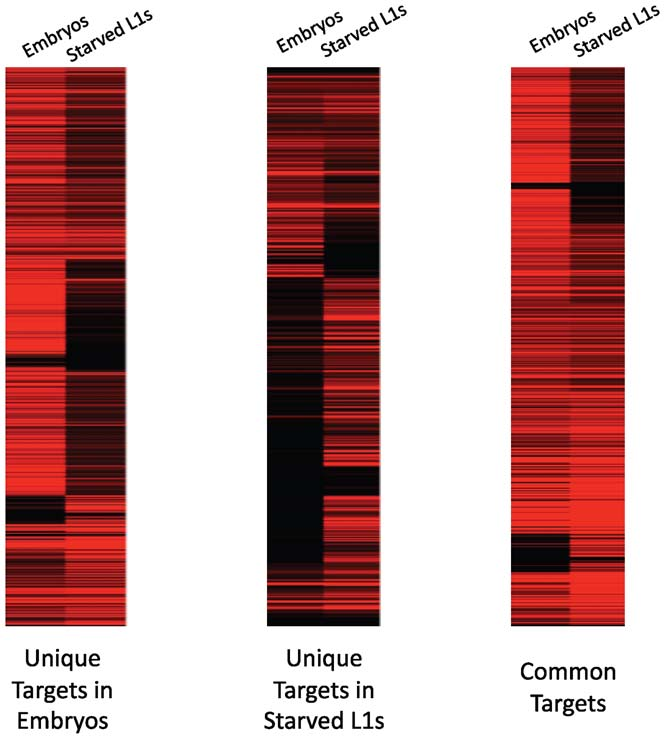
PHA-4 binding correlates with gene expression levels. The expression levels of PHA-4 targets show that binding correlates with increased gene expression. Genes bound by PHA-4 specifically in embryos tend to have higher expression (indicated by increasing red intensity) in embryos than in L1s, whereas genes bound specifically in L1s have higher expression in larvae than in embryos. Genes bound at both stages show a mix of expression levels.

We then correlated gene expression levels with PHA-4 binding using the RNA-sequencing data we gathered in embryos and L1 larvae. Overall, we found that 87% of genes bound by PHA-4 at either stage are expressed, indicating that PHA-4 rarely functions as a repressor at either stage. In support of this observation, we found that the expression levels of 74% of the embryo-specific PHA-4 target transcripts decreased in L1 larvae, when PHA-4 is no longer bound. The converse is also true: 69% of the L1-specific PHA-4 targets are expressed at lower levels in embryos, when PHA-4 is no longer bound (Figure 6). This finding indicates that PHA-4 might be directly involved in promoting the expression of most of its gene targets.

**Figure 7:**
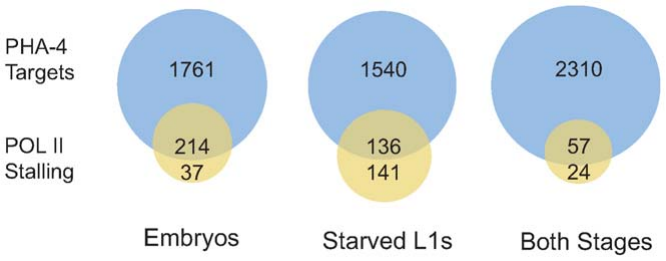
Genes displaying RNA Pol II stalling are preferentially bound by PHA-4. Pie charts showing the fraction of genes with an RNA Pol II stalling index >4 [25] bound by PHA-4 for genes with either stage-specific PHA-4 binding or shared PHA-4 binding.

Finally, we tested whether RNA Pol II “stalling” at transcription start sites (TSS) is affected by binding of PHA-4 in a stage-specific fashion. Stalling is the accumulation of RNA Pol II at the TSS, and has been experimentally defined as the presence of a peak of RNA Pol II binding at the TSS that is four-fold higher than binding within the gene body [25]. Stalling occurs preferentially at developmentally or environmentally regulated genes, presumably to hold RNA Pol II poised to respond rapidly upon the appearance of the appropriate cue. Stalling has been observed at ∼10% of genes in Drosophila and *C. elegans* previously [25],[26], but in our samples, we found that less than 2% of genes exhibited stalling in embryos and L1 larvae, likely due to experimental and culture differences (Dataset S4 and S5). However, PHA-4 binding clearly occurs at stalled genes at both stages more frequently than expected (Figure 7). Of the 277 genes with RNA Pol II stalling in L1 larvae, 49% are bound by PHA-4, which is twice the fraction of genes bound by PHA-4 genome-wide (23%). This effect was even more pronounced in embryos. Among the 251 genes with RNA Pol II stalling in embryos, 85% are bound by PHA-4, despite PHA-4 binding to only 20% of genes in the genome. This observation is consistent with the idea that PHA-4 regulates genes in response to developmental and environmental cues that influence the spatial and temporal regulation onset of gene expression.

## Discussion

We have established a pipeline to identify transcription factor binding sites in vivo in *C. elegans*. This pipeline is designed to take advantage of the stability of fosmid-based transgenes, as well as their reliability in reproducing native expression patterns. The transgenic lines emerging from this pipeline tend to have between one and three copies of the transgene, and exhibit minimal, if any, over-expression (Sarov et al., in prep). Our initial trials with the RNA polymerase II subunit AMA-1 indicate that the transgenic, tagged version of a transcriptional regulator can indeed successfully recapitulate the DNA binding properties of the native factor. This pipeline can now be used on additional factors, and because the same antibody is used for every immunoprecipitation, will provide fairly uniform investigation of the binding sites of multiple factors, and aid in the dissection of regulatory networks in development.

As a first step toward this major goal, we identified candidate gene targets of PHA-4 in vivo at two distinct developmental stages. We chose PHA-4 as the initial factor for binding site identification for three primary reasons. First, it is a well-characterized factor with fundamentally important, yet distinct, functions at different times in development. Second, a handful of direct transcriptional targets of PHA-4 have been independently identified and validated, providing some key positive controls. Finally, PHA-4, unlike AMA-1, is expressed tissue-specifically, primarily in digestion-related tissues such as the pharynx and intestine. Thus, it provides a test case for whether ChIP can be performed on transcription factors with restricted expression.

A little over half of the PHA-4 targets we identified are in common between these two stages, suggesting that PHA-4 does have a general function in regulation of gene expression. However, over 40% are preferentially bound in one stage relative to the other, indicating that the ability of PHA-4 to mediate different processes likely occurs through a shift in the sets of targets it regulates. These data indicate that transcription factors can have diverse and key roles in distinct biological processes and underscore the importance of identifying binding sites under multiple conditions.

Several interesting differences in PHA-4 binding were noted between the two stages. For instance, among the many examples listed, several genes encoding members of the dosage compensation complex were preferentially bound by PHA-4 in embryos relative to L1s. During embryogenesis, PHA-4 helps specify the pharynx at the same time that the dosage compensation complex (DCC) is beginning to implement a two-fold reduction of transcription levels from the entire X chromosome. Little is known about how the dosage compensation complex interacts with tissue-specific programs, and our data suggests that PHA-4 helps to control the levels of the DCC in order to provide more or less dosage compensation in that tissue as needed. Possibly, master regulators in other tissues also regulate DCC levels in order to bring the level of dosage compensation in alignment with the needs of a specific tissue.

We have also demonstrated a novel role for PHA-4 in promoting the survival of larvae during starvation. Reduced PHA-4 levels resulted in decreased survival, while conversely expression of PHA-4:GFP in a wild type background increased survival. In particular, the increased survival indicates that the role of PHA-4 in this process is a regulatable function. This function is in keeping with its noted role in regulating environmental responses, as well as controlling longevity and dauer formation [16],[18]. Identification of the PHA-4 binding sites under the starvation condition illuminates some aspect of this function. A quite striking increase in genes involved in fatty acid metabolism and sterol biosynthesis were seen in L1s relative to embryos. Accordingly, many nuclear hormone receptor genes, which encode proteins that bind steroid hormones, were preferentially bound by PHA-4. The nuclear hormone receptor gene family in *C. elegans* is much expanded relative to other organisms, and many of the ligands for these proteins are unknown. It is possible that a subset of these proteins respond to endogenous steroid hormones generated in response to starvation, and that PHA-4 mediates their induction.

Overall, the experimental ChIP-Seq pipeline we developed has produced global binding data, expanding the view of how PHA-4 works as both a master regulator of organ development and a mediator of starvation survival. PHA-4 primarily functions as an activator in both situations, based on our analysis of gene expression concomitant with binding analysis. It is likely that the different binding patterns of PHA-4 are mediated by potential cofactors such as SMK-1 [16], as well as interactions with other transcription factors such as the GAGA-binding protein suggested by the motif analysis here, and other studies [15]. The binding sites of these factors can be identified using the tagging system and experimental pipeline that we have established, and integrated with the PHA-4 binding data to understand the functional relationship of these factors. Ultimately, the global DNA binding datasets we gather will greatly facilitate formulation of developmental gene regulatory networks in *C. elegans*.

## Materials and Methods

### Clone construction and transgenesis

A 30–40 Kb fosmid containing the entire *pha-4* or *ama-1* locus, along with flanking regions, was selected from an available fosmid library (http://eleans.bcgsc.bc.ca/). Using recombineering [27], a tag containing GFP and three tandem copies of the FLAG epitope was engineered in frame at the carboxyl terminus of each gene. Additionally, the marker gene *unc-119* was placed into the backbone of the fosmid (Sarov et al., in prep). The fosmid clones containing the tagged genes were then prepped, and introduced into *unc-119(ed3)* mutant worms using microparticle bombardment [8]. Strains were tested for 100% rescue of the Unc-119 phenotype, indicating integration of the transgene. Integrated lines were then examined by fluorescence microscopy for expression of the tagged protein.

### Starvation assays

For starvation assays with PHA-4:GFP young embryos were released by bleach treatment and placed into modified S basal medium at 20°C (day zero). Three samples of 30 ul each (representing over 500 worms) were removed daily and plated with food to determine how many animals could mature beyond the L1 stage after two days incubation. For starvation assays using RNAi, embryos were incubated in dsRNA according to Ahringer [28] with the following changes. DNA template for dsRNA synthesis was prepared by PCR. Primer sets for GFP were 5′-TAATACGACTCACTATAGGAATTTTCTGTCAGTGGAGAGGGTG-3′ and 5′-TAATACGACTCACTATAGGTCCATGCCATGTGTAATCCCAG-3′ and amplified from bSEM538(pPD126.25). Primer sets for PHA-4 were 5′-TAATACGACTCACTATAGG-3′ and 5′-TAATACGACTCACTATAGGGATCCAACATCCATCACGACC-3′ and amplified from bSEM865. In vitro transcription was performed using PCR products as template with the Ampliscribe T7 Transcription Kit (Epicentre Biotechnologies). RNA was then treated with DNase and extracted using phenol/chloroform and ethanol precipitation. RNA was resuspended in a final concentration of 2 ug/ul.

Gravid hermaphrodites were bleached and embryos harvested and diluted to 100 embs/ul. In one PCR tube 2 ul RNAi soaking buffer (1.25×M9, 15 mM spermidine, 0.25% gelatin), 8 ul dsRNA at 2 ug/ul and 1 ul of embryo suspension was added. Three tubes per sample per day were prepared. Worms were incubated at 20°C for appropriate number of days. Day one was 24 hours after bleaching. To determine viability, three samples were taken and put onto plates with OP50 and are counted for worms bigger than L1 stage 2 days later. Variability in the number of worms per plate occurs because of pipetting variability, so numbers can go above 100% for one plate vs. the starter plate. The difference in buffers between the two types of starvation assays altered the survival times of worms in the two assays; animals incubated in RNAi buffer survived longer than in S basal.

### Strain growth, harvesting, and crosslinking

Liquid culture of worm strains was performed as described [9] with some modifications. Synchronized cultures of worms were grown on 10–20 150×15 mm plates until animals were gravid. The worms were then washed from plates using M9 buffer and bleached to obtain embryos. Embryos were transferred to 25–50 ml liquid media (S medium and nystatin), and incubated overnight at 20°C at 230 rpm rotation without food to obtain a synchronized first stage larval L1 culture. The worms were then transferred to 500 ml S medium with the anti-fungal nystatin and concentrated HB101, which serves as a food source. The worms were then grown at 20°C with shaking to the desired developmental stage before harvesting. Additional food was added as necessary. For starved L1s, PHA-4:GFP worms were collected after 6h without exposure to bacteria. To harvest, worms were centrifuged in 50 ml conical tubes at 3000 g for 2 minutes at room temperature. The worm pellet was then washed repeatedly with M9 buffer and centrifuged as before until bacteria were removed. If the sample was destined for IP followed by immunoblot, the pellet was directly subjected to this procedure (described below). If the sample was destined for ChIP-Seq, the sample was then resuspended in 47 ml M9 and 2.8 ml 37% formaldehyde solution, and crosslinked for 30 minutes at room temperature with rotation at 50–100 rpm. The worms were then washed with 50 ml 100 mM Tris pH 7.5 to quench formaldehyde solution, washed two times with 50 ml M9, and once with 10 ml FA buffer (50 mM HEPES/KOH pH 7.5, 1 mM EDTA, 1% Triton X-100, 0.1% sodium deoxycholate; 150 mM NaCl) supplemented with protease inhibitors (Roche Cat#11697498001, cOmplete Protease Inhibitor Cocktail Tablets). Worms were then collected in a 15 ml conical tube by centrifugation at 3,000g for 30s. The supernatant was discarded and the embryo pellet was stored at −80°C.

### Chromatin immunoprecipation (ChIP)

Chromatin immunoprecipitation was performed as described [9], with the following modifications. Approximately 0.5 ml of packed embryos/larvae was resuspended in 3 ml FA buffer plus protease inhibitors (2 tablets protease inhibitors, 250 ul 100 mM PMSF, 50 ul 1M DTT in 50 ml FA buffer). Using a Branson sonifier microtip, the sample was sonicated on ice/salt water 15 times at the following settings: 50% amplitude, 10 sec on, 59.9 sec off, avoiding overheating. Samples were transferred to microfuge tubes and spun at 13,000g for 15 minutes at 4°C. The protein concentration of the supernatant was then determined by Bradford assay. Extract corresponding to ∼2.2 mg of protein was added to a microfuge tube and the volume brought to 400 ul with FA buffer+protease inhibitors. Then 20 ul of 20% sarkosyl solution was added, and the tube spun at 13,000g for 5 minutes at 4°C. The supernatant was then transferred to a new tube, and 10% of the material removed and stored at −20°C for future use as input DNA. To the remainder, 15 ug of affinity-purified GFP (polyclonal goat IgG; produced in Hyman lab) or control IgG antibodies was added to the extract to detect the tagged transcription factor. Alternatively, 10 µL of mouse ascites containing the 8WG16 mouse monoclonal antibody was added (Covance, Cat. #MMS-126R) to detect RNA polymerase II. The immunocomplexes were rotated at 4°C overnight (16–20 h). Then 25 ul of protein A (anti-Pol II samples) or protein G (anti-GFP samples) conjugated to sepharose beads (Amersham Biosciences) were added to each ChIP sample and washed four times with 1 ml FA buffer, and spun at 2500g for 2 min to collect the beads. After the washes, the beads were suspended in one bed volume of FA buffer, and 40 ul of the bead slurry was added to each ChIP sample and rotated at 4°C for 2 h. The beads were then washed twice for 5′ each at room temperature in 1 ml of FA buffer and once in FA with 1M NaCl. Each wash was gently rotated, and beads collected between each wash by spinning for 1–2 minutes at 2500g. FA with 500 mM NaCl was then added to the beads and the beads were transferred to a new tube and rotated for 10 min. The beads were then washed in TEL buffer (0.25 M LiCl, 1% NP-40, 1% sodium deoxycholate, 1 mM EDTA, 10 mM Tris-HCl, pH 8.0) for 10 min and twice in TE for 5 min. To elute the immunocomplexes, 150 ul Elution Buffer (1% SDS in TE with 250 mM NaCl) was added and the tube incubated at 65°C for 15 min, with brief vortexing every 5 min. The beads were spun down at 2500g for 2 min and the supernatant transferred to a new tube. The elution was repeated and supernatants combined. At this point, input samples were thawed and treated with the ChIP sample as follows. To each sample, 2 ul 10 mg/ml RnaseA was added and incubated at room temperature for 1–2 hours. Then 250 ul Elution Buffer with 1 ul of 20 mg/ml proteinase K was added to each sample and incubated for 1–2 hours at 55°C, then transferred to 65°C for 12–20 h to reverse crosslinks. The DNA was then purified with the Qiaquick PCR purification kit (Qiagen), and eluted with 50 ul H_2_O. A 5 ul aliquot of the input DNA was then run on a 2% agarose gel to check the extent of shearing, with an expected range between 200–800 bp. The immunoprecipitated DNA was either interrogated by qPCR or subjected to high-throughput sequencing library preparation (below). All ChIP experiments were completed with two or more biological replicates.

### Immunoblot analysis

Immunoblotting was performed on worm lysates as well as immunoprecipited TF/DNA complexes. Immunoblot analysis of immunoprecipitated AMA-1:GFP and PHA-4:GFP was performed on non-crossed-linked worm lysates that had been subjected to the ChIP protocol until the multiple wash steps. Then 50 ul lysis buffer was added to the immunocomplex bound beads, and the beads were boiled for 5min before loading onto the gel. Ready Gel Precast Gels (4–15% polyacrylamide) from Bio-Rad Laboratories were used according to manufacturer's instructions. For AMA-1:GFP detection, anti-GFP goat polyclonal antibody was used, and for PHA-4:GFP detection anti-GFP from Roche (cat# 11814460001) was used, along with the species-appropriate secondary antibodies.

### qPCR analysis of ChIP products

To monitor enrichment of known or newly identified target genes, qPCR amplification of ChIP DNA was performed. Primers used are described in Table S1. Each PCR reaction of 10 ul was run through the following program in a Roche LightCycler 480 machine using the SYBR Green I Master kit (Roche 04 707 516 001) according to manufacturer's instructions. PCR program: Step 1: 95°C for 5 min; Step 2: 95°C for 30 sec; Step 3: 55°C for 30 sec, Step 4: 72°C for 1 min. Repeat steps 2–4 44×; Step 5: 72°C for 5 min; Step 6: 4°C.

### Library preparation for Illumina ChIP–Seq

The protocol for library preparation was adapted from the protocol “Preparing Samples for Sequencing Genomic DNA” by Illumina, and optimized with the following alterations. ChIP DNA was end-repaired using the ‘End-It DNA End Repair Kit’ from Epicentre, Cat#ER0720, then an ‘A’ base was added to the 3′ ends of the ChIP DNA using Klenow (3′ to 5′; NEB Cat# M0212s). The ChIP DNA was then ligated with the adapter mix from the Illumina kit using LigaFast from Promega (Cat#M8221). The DNA was then purified using the QIAquick PCR Purification Kit and protocol between each step. The DNA was isolated from a 2% Invitrogen E-gel (Invitrogen Cat# G5018-02) by cutting a gel slice between 150∼350 bp, which excludes adapter-adapters migrating at ∼120 bp. The DNA was then purified from the gel slice using the Qiagen Gel Extraction Kit, and subjected to PCR amplication with Phusion DNA polymerase (NEB Cat# F-531) and Illumina primers using the following PCR protocol: 30 sec at 98°C, [10 sec at 98°C, 30 sec at 65°C, 30 sec at 72°C] for 16 cycles, followed by 5 min at 72°C. The DNA was then purified on a QIAquick MinElute column and the 150∼350 bp band gel-isolated. Out of a 20 ul elution, 2 ul were used to measure the DNA concentration (ng/ul) and A260/A280 using a Nanodrop spectrophotometer. DNA with >5 ng/ul concentration is now ready for sequencing.

### RNA isolation and RNA–Seq

Worms were grown to the desired stage and pelleted as described above. Total RNA was extracted by TRIzol (Invitrogen) according to the manufacturer's protocol (TRIzol: pellet = 2∶1). PolyA RNA was purified using the Applied Biosystem (Ambion) MicroPoly(A) Purist kit. PolyA RNA was fragmented using Fragmentation Reagent (Ambion). First strand cDNA was synthesized from polyA RNA using a mixture of oligo dT and random primer (Invitrogen). Double stranded cDNA synthesis was performed using the SuperScript double stranded cDNA synthesis kit (Invitrogen). RNA-Seq libraries were prepared for sequencing using the Illumina protocol as described [29]. RNA-Seq scoring was performed as previously described [30]. The RNA seq dataset has been submitted to GEO (accession number GSE16552).

To assess the expression level of a given transcript, the DCPM (average depth of coverage per million reads) is calculated from RNA-Seq using a published method [30]. The change of expression level is determined by the DCPM of each transcript at different stages. The transcript with higher DCPM at a certain stage will be labeled as up-regulated gene at this stage.

### ChIP–Seq data processing and analysis

All mapping and analysis are based on genome WS170 of *C. elegans*. The annotation of the genome includes 27,322 transcripts (20,084 genes), which were confirmed from a previous study [30], where most of the transcription start sites (TSS) were defined. If no TSS was found, it was set as 150 base pairs upstream of the ATG site.

Raw data from the Illumina Genome Analyzer I and II were analyzed with Illumina's Firecrest, Bustard and GERALD modules for image analysis, basecalling and run metrics respectively, and a PhiX174 control lane was used for matrix and phasing estimations, as per the manufacturer's instructions. Then, the sequence reads were mapped to the *C. elegans* genome using Illumina's ELAND program in standalone mode. For each sample, the numbers of total and mapped reads were determined (Table S5). ChIP-Seq with two separate biological replicates with either the anti-GFP antibody (Germany) or anti-Pol II antibody (Clone 8WG16, Covance Research Products Inc) were pooled together for signal calling. Significant “ChIP hits” were created using a 200 bp sliding window and scoring was performed with the PeakSeq program [20]. The hits were further filtered by using various p-values of PeakSeq (Figure S7).

The Integrated Genome Browser (IGB, Affymetrix) was used to view images of signal tracks and to overlay them onto the *C. elegans* genome. Each GFP and POL II sample was compared over input DNA signal. To build signal tracks for comparing samples with different number of sequencing reads, the y-axis was normalized for each sample according to the total number of mapped reads.

In order to show the concordance of two antibodies (anti-GFP and anti-Pol II) for the AMA-1 binding experiments, we compared the hits from PeakSeq with p value cut-off 0.001. Every PeakSeq hit was divided into 600 bp bins. Then the tag count of each bin was normalized against its background input. The normalized tag counts of two antibodies were correlated significantly (average correlation coefficient, R, is 0.934; Figure 2B).

To determine which genes showed elevated Pol II or GFP signal over TSSs, we bypassed the first pass of Peak-Seq that determines potential binding regions by simulation. Instead, we directed Peak-Seq to examine 24,678 regions corresponding to TSS sites with a 300 bp pad on each side of the TSS. Peak-Seq was then used to determine whether these 600 bp regions were enriched relative to input DNA.

All ChIP-Seq datasets have been submitted to GEO (accession numbers GSE15535, GSE15628, GSE14545), and all tracks are available for viewing at the modENCODE website (www.modencode.org).

### Target finding

The high genic density of the *C. elegans* transcriptome makes it often the case that several genes are within a few kilobases of a binding site, and it is necessary to select the most likely targets amongst them. We therefore wrote an algorithm that first searches for all transcripts within 5kb of the midpoint of a binding site. The distance between the binding site and each transcript is computed as one of three possibilities: binding site is upstream a certain number of bases from the TSS, downstream a certain number of bases from the TES, or within the gene. Transcript isoforms are then grouped into genes assigning the distance to it as that of the closest isoform. The genes are then ranked by the likelihood of being the target according to the following criteria: most likely target is that which has binding site within it, next most likely is that which is downstream of the binding site (if multiple targets are downstream they are ranked by their distance), and the least likely are those that are upstream of the binding site (if multiple targets are upstream they are ranked by their distance). The targets are then grouped into the following four bins: (1) target genes which have an internal binding site or that are less than 2kb downstream of the binding site, (2) targets that are within 2kb–5kb downstream, (3) targets that are less than 2kb upstream of binding site, and (4) targets that are within 2kb–5kb upstream. Finally, the binding site is said to target all genes in the first non-empty bin. Examples of how assignment of a binding site to candidate target genes occurs are shown in Figure S3.

### Pol II stalling

To determine whether Pol II is stalled in a gene, we created a differential signal map by subtracting the tag count of the factor from that of the input at each position. This map was used to calculate the average tag count for promoter regions and over the bodies of transcripts. For this analysis, the promoter region is defined as ±300 bp from the TSS. The transcript body is the region 600 bp downstream of the TSS to the end of the transcript. If the ratio of promoter∶body average transcript count is greater than 4, Pol II is considered stalled. For lower ratios, Pol II signal is deemed to be either uniform or absent depending upon whether Peak-Seq detected Pol II enrichment in the transcript. This is the same method used by Zeitlinger et al. [25].

### Gene Ontology (GO) analysis

A stringent q-value cut off defined by PeakSeq, 1×10^−5^, was used to define the genes targeted by PHA-4 in embryos and starved L1 stages (Figure S7). The binding site has to be within or upstream 2000 bp of the targeted gene. GoStat (http://gostat.wehi.edu.au/cgi-bin/goStat.pl) was used for finding the over-represented and under-represented GO terms [Table S2 (embryos) and Table S3 (L1)]. GO categories were taken from the “biological process” level. 1975 and 1676 unique targeted genes at embryonic and L1 stages are analyzed respectively, of which 1328 and 905 are annotated in the GO database for embryonic or L1 stage. The top ten enriched GO terms at each stage are listed in Figure 3C and 3D.

### Gene Set Enrichment analysis

The same target list for GO analysis was used for Gene Set Enrichment Analysis (GSEA) [31] in embryos. An expression dataset of 8,769 genes were analyzed in a previous microarray study on pharynx development in embryos, comparing two mutants, *par-1* (excess pharynx) and *skn-1* (no pharynx) [14]. Of these, 2348 are defined as PHA-4 target genes from our embryonic target list. GSEA [31] (http://www.broad.mit.edu/gsea/) showed significant enrichment of these targets among the up-regulated genes, which means they are more highly expressed in *par-1* embryos with excess pharynx, than in *skn-1* embryos that lack pharynx.

### Motif analysis

The motif analysis was performed by MEME (http://meme.sdsc.edu/). MEME [32] was used to discover the motifs and generate the position weight matrices (PWMs) for PHA-4 in embryos and starved L1s. For the embryonic stage, the input data to MEME was the central 200 bp corresponding to the center of the peak of the bound region. All the input sequences were sorted by their p-values reported by PeakSeq and the top 200 sequences were chosen for motif discovery. For the L1 stage, sorting by p-values failed to find a significant match of the known PHA-4 consensus motif, because of a higher signal from the input sample. Instead, sequences were sorted by their signal ratios over input and the top 200 sequences with a more stringent window of 100 bp were chosen.

To calculate the enrichment of the observed consensus motif, MAST [32] was used to search for sequences that contain the motif represented by the PWMs generated by the aforementioned. The input data to MAST was the central 1000 bp corresponding to the peak. All the sequences were sorted by their p-values and the top 200 sequences were chosen. For the background, 1000 bp was taken from 1000 bp upstream of the central for each sequence with p-value<0.05. The p-value cutoff for each motif match was <0.0001. The enrichment was calculated by comparing the number of sequences matched the motif in the bound regions to that in the background regions. The p-values of enrichment in both embryos and L1s are close to 0.

## Supporting Information

Figure S1 Characterization of AMA-1:GFP expressing animals. (A) Transgenic animals express AMA-1:GFP in all nuclei, recapitulating the wild-type expression pattern of RNA Polymerase II. (B) Immunoprecipitation followed by immunoblot analysis. AMA-1:GFP can be immunoprecipitated from worm lysates with anti-GFP (GFP IP), which recognizes the tagged AMA-1, and anti-Pol II (Pol II IP), which recognizes both tagged and native protein. However, immunoblotting was performed with anti-GFP goat polyclonal antibody, so native Pol II is not detected. Control IPs include goat IgG (gIgG) and mouse IgG (mIgG). Total lysate (input) was included as a control. (C) Correlation analysis of two biological replicate ChIP-Seq experiments immunoprecipitated with anti-GFP and anti-Pol II antibodies show high correlation between the two IPs. (D) Correlation analysis of two biological replicate ChIP-Seq experiments for PHA-4 in embryos and starved L1s.
(3.76 MB EPS)

Figure S2 Examples of AMA-1:GFP and RNA Pol II binding at individual loci. ChIP-Seq data acquired with anti-GFP antibody is shown in green, anti-Pol II is shown in red, and the input signal is shown in blue. (A,B) AMA-1:GFP and native RNA Pol II binding at the promoters of ubc-3 and wwp-1. (C,D) AMA-1:GFP and RNA Pol II bind throughout the gene bodies of unc-108 and ftp-1. (E) Possible accumulation of binding at the 3'end of sulp-6. (F) Y71G12B.6 does not display detectable AMA-1:GFP or native RNA Pol II binding.
(4.44 MB EPS)

Figure S3 Diagram of how gene targets were defined. Demonstration of how algorithm calls targets for a given binding site. The red rectangles depict binding sites found by the PeakSeq algorithm. Binding site A was assigned to two gene targets rfc-4 and eft-3 but not F31E3.2 (black) since it is greater than 2kb away from the site. Binding site B was assigned only to F31E3.4.
(0.75 MB EPS)

Figure S4 Genes expressed in the pharynx are preferentially bound by PHA-4 in embryos. Gene Set Enrichment Analysis (GSEA) [27] shows that PHA-4 target genes at the embryonic stage are highly enriched for genes up-regulated in animals with excess pharynx relative to no pharynx [14]. 2,348 of the 8,769 genes surveyed in the previous microarray study [14] are bound by PHA-4. The ranked expression values (log2 of par-1(excess pharynx)/skn-1(no pharynx) of all 8769 genes are plotted from left to right at the lower panel. The upper panel is the enrichment score (ES), a running sum statistic beginning from the highest ranked gene at left. ES increases when a PHA-4 target is encountered and decreases otherwise. The ES of a set of 2,348 randomly selected genes from the 8,769 set is also presented as the dotted line under the ES of PHA-4 targets. The normalized enrichment score of 2348 PHA-4 targets is 2.23, with q-value less than 0.001.
(0.86 MB EPS)

Figure S5 The PHA-4-binding consensus sequence is enriched under PHA-4 binding peaks. The top three motifs are listed for embryos and starved L1s separately. In embryos, the most conserved sequence is GAGAGAS (S = [GC]). The second most conserved sequence is TGTBTSY (B = [TGC],S = [GC],Y = [TC]), which is compatible with the published motif, TRTTKRY (R = [GA],K = [GT],Y = [TC]). The reverse-complementary sequence of TGTBTSY is shown in the figure of embryos. The enrichment of this motif in embryos is 3.2. In starved L1, TRTTKRY compatible motif is ranked at the top, with an enrichment of 2.4.
(1.04 MB EPS)

Figure S6 Examples of PHA-4 binding sites at individual loci. Genes associated with autophagy are shown. ChIP-Seq data acquired with anti-GFP antibody is shown in green and the input signal is shown in blue. (A–C) PHA-4:GFP binds at the promoters of bec-1, lgg-1, and unc-51 at a very high level in starved L1 animals as compared to its binding at the same promoter regions in embryos. (D) PHA-4:GFP binds to the promoter of gpd-2 in starved L1 animals, but it no longer binds to that promoter in embryos.
(3.51 MB EPS)

Figure S7 Binding site and gene target identification at multiple cutoffs. Chart displaying the number of binding sites and targets as a function of p-value determined by PeakSeq. Genes are defined as PHA-4 targets if the binding site is within the gene or less than 2,000 base pairs upstream to the TSS (transcription start site). At each p-value cutoff to define binding sites, the target calling algorithm was run and the number of total targets for both embryo and L1 samples computed. At stringent cutoffs between 1×10^−6^ to 1×10^−3^, each order of magnitude change in the cutoff changes the number of targets called by about 500. The number of binding sites also changes by about the same amount. For the analyses in this paper, a p-value of less than 1×10^−5^ was selected unless otherwise specified.
(0.32 MB EPS)

Table S1 Primer sets and fold enrichment of PHA-4 binding sites.
(0.14 MB DOC)

Table S2 GO analysis of unique PHA-4 target genes in embryos.
(0.11 MB DOC)

Table S3 GO analysis of unique PHA-4 target genes in starved L1s.
(0.04 MB DOC)

Table S4 Individual functional analysis of subset of named genes.
(0.06 MB DOC)

Table S5 Total number of mapped reads of ChIP-Seq experiments.
(0.03 MB DOC)

Dataset S1 Complete target list of PHA-4 in embryos.
(0.04 MB XLS)

Dataset S2 Complete target list of PHA-4 in starved L1s.
(0.04 MB XLS)

Dataset S3 List of subset of named gene targets.
(0.04 MB XLS)

Dataset S4 Complete target list of POLII stalling in embryos.
(0.00 MB XLS)

Dataset S5 Complete target list of POLII stalling in starved L1s.
(0.00 MB XLS)
