# Supplementary material for: Genome-Wide Identification of Binding Sites Defines Distinct Functions for Caenorhabditis elegans PHA-4/FOXA in Development and Environmental Response
Source: PLoS Genet. 2010 Feb 19;6(2):e1000848. doi: 10.1371/journal.pgen.1000848 (PMC2824807; doi:10.1371/journal.pgen.1000848)
Supplement: Table S1 — Primer sets and fold enrichment of PHA-4 binding sites. (0.14 MB DOC) [file pgen.1000848.s008.doc]

**SUPPLEMENTARY TABLES**

**Table S1. Primer sets and fold enrichment of PHA-4 binding sites**

| **Oligo Name** | **Forward Sequence** | **Reverse Sequence** | **fold enrichment** | |
| --- | --- | --- | --- | --- |
|  |  |  | **embryo** | **starved L1** |
| Fer-1 (I:8630462-8630440) | GCTTTGGAAAACGGAAACTGCAG | ATTTTGTCGCCACTTTGGAATTG | 1.00 | 1.00 |
| II:9577607-9577738 | AAACGTTTGCATATATTTCATTTCA | CAAGTCTATATGGAAGAGATGGAGC | 3.20 | 1.60 |
| V:532012-532235 | ACAACCAGCATAATTGTTCGAAAT | ATTATTTAGGGGGAATACCATTGAA | 3.75 | 3.10 |
| I:9773963-9774159 | ACAGCACGGTTTTTGACAGAATA | TTGATCAAGAGAAAGAGTATGGAGC | 2.00 | 2.40 |
| V:10807895-10807998 | AGTGGCTCAAGACTATCTTTTTGG | TTGTATCATTGTTTTTGTTTCTCCA | 5.39 | 2.60 |
| V:9288820-9289067 | ATCTGGTCGAGTGTCTCATATTCA | TGATTATCACTTCCTTTTACGCTTC | 3.62 | 2.90 |
| V:12184332-12184460 | ATTGGAACGTGCGCATTCAG | AACTCAGTTTTTCAGTCATTTTTCG | 2.14 | 4.00 |
| I:13028431-13028548 | CACAAAATGAGCCACAGGAGAT | TGAAAATGATGAGTAAATTGGGATT | 4.12 | 3.20 |
| IV:15312024-15312181 | CACACTTTCAGTTATAAATTCACTGC | TACATCTTCTCACTGACTCGTTGAC | 2.18 | 3.20 |
| X:8736981-8737119 | CAGACCGAAAATCAATAGAAAAACC | TGACAGTTTAAAAATGGTCTGTGAA | 10.88 | 3.50 |
| I:6535289-6535437 | CCCATATCTATAACAAAAAGGATATGAA | ATTTCAAGTGCTCTTGTTTTGTTTC | 3.02 | 1.40 |
| IV:4672760-4672912 | CGCATAGTCACCATAGTCAAAACT | ACAACTGAGTCTCTTCACCGATTAC | 1.96 | 2.90 |
| III:95005-95184 | CGGAATCTATAATTTCAAGCCAAA | CTGCGTCTCCTACATAGTCCTCTAC | 4.63 | 4.30 |
| III:10848737-10848837 | CTCCTTCTAATCTCACACAATCTTGA | CGAAAATTAATGTTTGCATCAAAGT | 4.03 | 2.50 |
| V:13041665-13041813 | GATCCCGTTGGAAGAAGGAT | CCCCTTTTTCTATTAAATGTGTTCA | 1.33 | 3.30 |
| III:5591751-5591860 | GCAGGATAAATGGATTAAATGTATGA | ATATTCTGCGAAATTCTGTCTTTTG | 4.05 | N/A |
| V:19153232-19153380 | GCCTAAAAAGCATTTCTTTAGTGGT | AGAAACATAATACCTAGGGGACACC | 0.32 | 1.80 |
| I:6780061-6780256 | GCTTATGAAAACAAAACAAAAACAA | GTCTTACCACGATCTCATGTTTCTC | 2.63 | 6.70 |
| II:15264574-15264785 | GTATATTGCCATAGCCACCACC | AGTGGGTGGTATGTATTTCTTTCAA | 2.06 | 3.70 |
| I:6105632-6105740 | TGCAATAAATGATTTTCAAACAAAA | AGAGTTATCCCTGTCTGAATGATTG | 2.02 | 2.30 |
| IV:2725835-2726071 | TGGAAAATTGAAGGTTTTCACTGTA | TGTTGATTTATTATCAATTCGCAGA | >4.7 | 3.40 |
| I:9505983-9506154 | TGTATTTTTCTTGTTAATTCTCGCTC | ATATTTTGAGACCCGAAGAACTCTG | 2.26 | 2.80 |
| III:13296253-13296440 | TGTTGGAAAAAGAAATAAATAAATACGA | AAACTGTTCTATTCTAATCGAGGGG | 3.68 | 5.30 |
| I:9422207-9422423 | TTATAGAATAAACAATGGGGAAAAGG | AAAACTGCGGTAAAGTTGATTAGTG | enriched* | 2.00 |
| IV:10066485-10066615 | TTATCTTCGGTTTTCATAATATCAG | CACATGCAAACGTCTTATCACTCTA | 1.39 | 1.07 |
| III:3065967-3066170 | AAAAACCGAGAGAGTGGAGAGAC | ACCCCGTAGACTTTTGAACTTTACT | 1.74 | 5.02 |
| IV:4093131-4093337 | AAAGTTGCAGTGCCAAATGAGT | AGATAGTGCGGAAAATTAGGAGAGT | 1.94 | 3.12 |
| X:14024465-14024634 | AATCAATTGAACAGATTGGCGTA | AATAATACACACACAGACAACACGC | 1.39 | 1.94 |
| I:12826579-12826748 | AATCCAAATTTCCACGAGTTTTT | TAGAGAGAGAGAAAGAGACGACGAG | 2.03 | 3.50 |
| IV:13881768-13881910 | ACAATTTAACAAACAATTTTCAAAG | GAGAGAGTGTGTGAAAAGAGGAGAC | 2.66 | 3.87 |
| I:9042809-9043034 | ACACATGAAATGAACAAGAAGTGCT | TTAAATGGCATTGTTTCTCTAAACC | 2.04 | 1.94 |
| III:8416926-8417173 | ACACGTCTCTTCCGCCACTT | AAACGAAGAGAAAAAGAAAGAGAGC | 2.38 | 2.61 |
| III:13186817-13187049 | ACTTAGATATTCGGAATCAGGGAGT | AAGAAGAGAGCATAGAACAGAACCA | 3.01 | 2.43 |
| V:17870585-17870727 | AGAAGCTCGTTTTGCATCTTTTT | CCATTGAGAACGACTGTTTATTTGT | 3.17 | 2.91 |
| X:13060003-13060199 | ATCGGTGTGGTTTTCAATTTTT | CGTGCTTTAGATAAGGGTGAAATAA | 1.86 | 1.55 |
| I:7480059-7480175 | ATTTCTTTGGTATCCATGGGC | ATTGGAAAAATCCTTAAAAATCTGC | 3.43 | 0.38 |
| III:3887583-3887683 | ATTTGAGCACCAATTCGAGG | AGAGTGTGTAAGAGAAACAGGGAGA | 1.57 | 2.00 |
| III:2375909-2376114 | ATTTGTTTTCCAGGGGCATATCT | ACGCAAATTAGTTTGAAAATGAGAC | 2.41 | 1.93 |
| IV:12548325-12548449 | CAAACATTTGAAAAAGAGCCCTA | CGATTAATCTCTCTCTTAGCCACAA | 3.36 | 0.79 |
| IV:126121-126343 | CAGCTGGAATAGAACAGACAGTACA | AGTACCGAGTACGGTCAACAATTTA | N/A | N/A |
| X:16681702-16681867 | GCTAGAAAGGACTAGACCAACCAAT | AACAGCATTTGAAGTGATACTGCTC | 2.05 | 2.78 |
| III:10218437-10218585 | GTTCTGTTTCTTGGCGTTTTGTTAT | TAAACGAGATCCGCTATTATTTTTG | 3.17 | 1.29 |
| II:14528246-14528475 | GTTTCTTGAGGAGACCTCACAACT | CACACCTAACATAACCCTGAGAAAT | 7.82 | 4.46 |
| X:11597650-11597837 | TAAACGAAAATGCAAAAACAATGAT | TGAAAACAGGGAGATAGAGAGAGAA | 2.27 | 2.47 |
| II:14349424-14349559 | TGCTTTTTAACGATTTAAACAATTT | CGTAGTCTCCAAAACAATTAAGCAT | >1.9 | >2.7 |
| X:8623894-8624057 | TTGAACTACTTGCTAGGTTACAGTTG | CAACAAATTGTCCATCATTTCTCTA | N/A | 0.88 |
| IV:2388920-2389129 | TTGGGCCTAAAATATCTTCAAAAA | ACACATAAGGGGTACACACGAAAT | 3.06 | 5.91 |
| III:517474-517657 | TTTCCTAATCAAGAGGTCATTGAAG | AAGAAGTATATTGTTTGTTGGACGC | 3.05 | 1.00 |
| I:5244179-5244347 | TTTTACTGAAAACCCATGTTAAAAG | TAGAATATTTTGGAGAAAACCTTGC | N/A | N/A |
| III:6606133-6606281 | AAAAATAAAATCATCAGCGTTTT | TGATGAACAGAGTTACAGAGAGACG | 4.30 | 2.01 |
| V:15016807-15016936 | AAAAATGACACAAAACACAAAAA | CATGACTCTGAAGGTAAACACACTG | 4.10 | 1.89 |
| X:12670315-12670499 | AAAAGATAGACGAAGTAGAAAATGGTG | AAGCAAGTTGTTCTCAACTGTCACT | 2.50 | 5.05 |
| IV:15561119-15561304 | AAAATCCATAAAACTGTTTGACTATTGA | TAGTGTGCAAAGTGAAGAGAGTGAG | N/A | N/A |
| V:10178227-10178377 | AGTTTCGGGGACATTTGAGTTT | GCAGATTCATGAAGAAATGATACAA | 2.90 | 4.09 |
| IV:13980145-13980294 | ATTGAAACATGAAAAGTGCGTGT | TCAAAACATGACAAGTAGTTGAACG | 5.40 | 2.75 |
| V:10691328-10691513 | ATTTTGCCATCGGTCTTCATT | GAAGAACAAATATCTGCGTCTCACT | 3.70 | 3.91 |
| X:3224815-3224929 | CGAGGCGAGTGCTAAGTTAAG | CTTGTCGTATTGTTTGGAACTTTCT | 1.40 | 2.56 |
| IV:10502330-10502442 | CGATGTCATTTTCTAACTACTGTTCAA | CCATTACTTTTGATCTTTCCCTTTT | 4.40 | 1.36 |
| X:10637072-10637255 | CTAAGGGGCATGTGGGTCTA | ATACATTCAGAAAGATGACAGAGCC | 1.40 | 2.65 |
| II:155955-156122 | CTCGCCGAACGTTTGGAGTTA | TACAGATTAGGGAAACACGCTCTAC | 1.20 | 7.06 |
| X:1089989-1090134 | CTGACTCTCACAACAAAAGGAACTT | ATGCTTTAACCCCTTTGCTACTACT | 2.70 | 3.12 |
| I:12547811-12548028 | CTTAACAAAAACCAGTTGGAAACC | TAGGGGGAACTTGTTAGAATTTTTC | 9.80 | 4.86 |
| II:11979566-11979816 | CTTCGGACCTGAAAGTCTTGATT | AAAAGTTTCGTATAATCGTGTTTCG | 9.90 | 4.32 |
| I:11194925-11195151 | CTTCTTTCCAACATCATCTAACACA | AACAGTCCGTTTTCACAAGAAGTAT | 2.60 | 3.03 |
| II:11305397-11305506 | GGTTCCCAGTGCTCTTTTACTATTT | TAACAACGAAGTGAGATCATCAGAG | 7.90 | 3.02 |
| X:9551031-9551213 | GTAAAAGTGCACTCCGACACTG | AAGGCGTTAGAAGAATGAGTGACTA | 2.00 | 8.94 |
| II:10382154-10382269 | GTGTCGCCAAAAAGATTTCCTAC | TCTTATTGAACTTTTGTGTGTGTGC | 47.80 | 0.29 |
| X:7151023-7151176 | GTTTCCGTCGGCTCCAAAAT | CACACTCAACAGATAGTCGGTACAC | 1.90 | 1.58 |
| V:6043686-6043924 | TCAACACAATAGCTTTGATAAGGAA | CAAAATAAACATTTGGAATTTCAGC | 14.00 | 5.04 |
| I:9331315-9331549 | TGTTGCAAAACTTTCCAAAAAT | CTCAGACAGAGAGAAGTGTTGTTGA | 8.20 | 7.18 |
| I:6883399-6883516 | TGTTTCAACTTTCTTTCTCTGAACG | ACAAAAGTGGTAGCATTCTTCAGTC | 1.30 | 5.13 |
| IV:8199308-8199467 | TTGAGAAATATCGTAAAAGGCAAAC | TTTTTAAATCGAAATCTCGACACTC | 3.80 | 1.84 |
| X:6593419-6593533 | TTTTTCCTTCACGCATTGCT | TAAACCATAGACATTCCTCTTTTCC | 3.50 | 2.99 |
| III:3689410-3689557 | AAAAATGGCTTGTGAAACATGG | AGTTCAATTGACGTTCGTTTTAATC | 1.90 | 2.65 |
| V:8548889-8549119 | AATTTGATGATTCTCCCACTCAAT | CTTATTCTCTCCCTGATAAAGCTCC | 15.20 | 2.04 |
| III:5552897-5553094 | ACTACCGTAACTCTTTAAAGGCGCA | CTGACAATTTGTTTACCTTGGAATC | 9.80 | 6.38 |
| V:18669695-18669804 | ATTTGGTGGGTGTTGTCGAT | ATGTCTATTTCTGTAACTTCGGTGC | N/A | N/A |
| V:13777821-13778019 | CGCCTATGGACGGGTACTAAAT | GCATTTGAACTTTCTTTTTGTCAAT | N/A | N/A |
| II:1525373-1525618 | CTTAACCATAGTTCCGCCAAACAC | AACAAGTAGTTGGAACGAGTGGTAG | 2.80 | 2.27 |
| III:2760719-2760842 | GAGGAATAATAGTCGGTAGATCATTTT | TCTGTACTATAAAACGACGCTTTGG | 3.00 | 2.05 |
| III:2719567-2719759 | GCAGAAATCAATTTTCTCGTAGATTT | TTAAACAAATAGATTGTCCGAAAGG | N/A | >4 |
| IV:3813959-3814067 | GGAGTCCACGGAAGAATACTCTAAG | CGTGGAATAAATTTCAACCAATAAG | 2.40 | 5.05 |
| III:4440045-4440292 | GTAGTTTTTGACTATTTCCCCACAG | AAGAAAGAATGAGGAAATGTGTTTG | 3.80 | 2.22 |
| V:12906492-12906729 | TTTTCGGAAGGCAATTTCATAG | ATCATAGTTTAACACGTCATGGTCA | 1.90 | 4.36 |
| II:9970575-9970792 | TTTTTCTCAAATGAAAAGAAATTG | ATTTTTCACATACACAACTTTTCCC | N/A | N/A |
| V:11339866-11339992 | AAAACCTGACTATTGCTTGGTTATC | GCGTGATTGAGATAGGTAAAGGTAA | 3.70 | 1.48 |
| X:2367220-2367350 | AAATAATAAGAATGGATGTTTTCCA | TCTTGTTTGACCAAGCTCTTTATCT | N/A | 2.76 |
| V:9289874-9289990 | AAATGTGCGAAACTGTAAAGATGAG | AATCTGGCATACGGAAGAATACTAA | 3.10 | 1.91 |
| I:7790867-7790984 | AAATTAACAAATTCCAAAAGAGCAA | ATACTTGACTGACTACAAGCCGAAC | 4.70 | 1.73 |
| II:11567067-11567263 | ATTAGTACGGTAAGAGCAAGCAGAA | AGAAGACCTCGTCTATTGGTGAAAG | 2.20 | 1.39 |
| III:4037286-4037509 | ATTATAACAGGCGGTGCATTCTAGT | CTCTTCAAACTCTAACCATCTCTCG | 8.70 | 2.02 |
| X:2885315-2885447 | GACCATTGGGAGATGAAAACC | GTGTGTGTGTGTGTGTGTGTCTACT | 1.20 | 1.77 |
| I:2831401-2831578 | GAGGATAGTGGAACGAGGAAAAC | AGAGACGCAGAGAAATTGGTGTAG | 1.40 | 2.79 |
| III:4666670-4666905 | TTCTCATGGTCAAATGTCGGT | AGAAATTTGAGAGAGAGAGGGAGAG | 1.20 | 2.40 |
| V:11203010-11203209 | TTCTTTGTTTGTAAACTCTTTATTTTCC | AGGACAAGTGGTACTTGAGTAGTGG | 3.90 | 2.04 |
| Fer-1 (I:8630287-8630309) | GCTTTGGAAAACGGAAACTGCAG | ATTTTGTCGCCACTTTGGAATTG | 1.00 | 1.00 |

N/A means no products after qPCR cycles.

enriched* means we did not determine an accurate fold of enrichment by calculating the Cp value, but the enrichment is confirmed by running the qPCR product on a gel.
