# Supplementary material for: Genome-Wide Identification of Binding Sites Defines Distinct Functions for Caenorhabditis elegans PHA-4/FOXA in Development and Environmental Response
Source: PLoS Genet. 2010 Feb 19;6(2):e1000848. doi: 10.1371/journal.pgen.1000848 (PMC2824807; doi:10.1371/journal.pgen.1000848)
Supplement: Table S2 — GO analysis of unique PHA-4 target genes in embryos. (0.11 MB DOC) [file pgen.1000848.s009.doc]

Table S2. GO analysis of unique PHA-4 target genes in embryos

| **ID** | **Name** | **Fold** | **p-value** | **Count** | **Database**  **Count** |
| --- | --- | --- | --- | --- | --- |
| GO:0009790 | embryonic development | 2.0 | 0 | 581 | 2752 |
| GO:0007275 | multicellular organismal development | 1.9 | 0 | 696 | 3542 |
| GO:0009791 | multicellular organismal development: post-embryonic development | 2.1 | 1.2E-63 | 389 | 1744 |
| GO:0048513 | multicellular organismal development: system development: organ development | 2.5 | 1.5E-40 | 184 | 707 |
| GO:0048731 | multicellular organismal development: system development | 2.4 | 1.6E-39 | 191 | 755 |
| GO:0003006 | reproductive developmental process | 2.5 | 3.6E-35 | 157 | 597 |
| GO:0048856 | anatomical structure development | 2.0 | 1.1E-33 | 253 | 1197 |
| GO:0040009 | regulation of growth rate | 1.9 | 1.1E-32 | 270 | 1324 |
| GO:0045927 | positive regulation of growth | 1.8 | 2.7E-32 | 303 | 1556 |
| GO:0016043 | cellular component organization and biogenesis | 2.1 | 2.7E-30 | 197 | 879 |
| GO:0051301 | cell division | 3.2 | 8.2E-28 | 77 | 230 |
| GO:0007610 | behavior | 1.9 | 1.3E-27 | 247 | 1240 |
| GO:0040016 | embryonic development: embryonic cleavage | 3.1 | 6.4E-22 | 63 | 191 |
| GO:0050789 | regulation of biological process | 1.5 | 7.8E-21 | 422 | 2686 |
| GO:0019953 | sexual reproduction | 1.9 | 5.1E-17 | 152 | 751 |
| GO:0009653 | anatomical structure development: anatomical structure morphogenesis | 1.9 | 1.1E-15 | 150 | 757 |
| GO:0051641 | cellular localization | 2.3 | 3.1E-14 | 82 | 343 |
| GO:0051649 | establishment of cellular localization | 2.3 | 1.2E-13 | 80 | 337 |
| GO:0033036 | macromolecule localization | 2.4 | 2.1E-12 | 62 | 244 |
| GO:0045184 | establishment of protein localization | 2.3 | 1.8E-08 | 48 | 199 |
| GO:0040014 | multicellular organism growth: regulation of multicellular organism growth | 1.8 | 5.4E-08 | 92 | 490 |
| GO:0035264 | multicellular organism growth | 1.8 | 5.4E-08 | 92 | 490 |
| GO:0044237 | cellular metabolic process | 1.2 | 1.8E-07 | 458 | 3495 |
| GO:0044238 | primary metabolic process | 1.2 | 7.4E-07 | 461 | 3555 |
| GO:0008406 | multicellular organismal development: development of primary sexual characteristics: gonad development | 2.9 | 1.2E-06 | 22 | 71 |
| GO:0043170 | macromolecule metabolic process | 1.2 | 5.8E-06 | 392 | 3002 |
| GO:0051656 | establishment of organelle localization | 2.5 | 6.2E-06 | 27 | 102 |
| GO:0045137 | multicellular organismal development: development of primary sexual characteristics | 2.7 | 1.6E-05 | 22 | 78 |
| GO:0022402 | cell cycle: cell cycle process | 2.0 | 2.1E-05 | 45 | 216 |
| GO:0007049 | cell cycle | 1.9 | 2.7E-05 | 49 | 244 |
| GO:0009886 | multicellular organismal development: post-embryonic development: post-embryonic morphogenesis | 1.7 | 3.4E-05 | 66 | 362 |
| GO:0019098 | reproductive behavior | 1.8 | 5.2E-05 | 56 | 296 |
| GO:0048609 | multicellular organism reproduction: reproductive process in a multicellular organism | 1.8 | 8.5E-05 | 56 | 300 |
| GO:0032504 | multicellular organism reproduction | 1.8 | 8.5E-05 | 56 | 300 |
| GO:0007059 | chromosome segregation | 2.3 | 1.5E-04 | 24 | 97 |
| GO:0009058 | biosynthetic process | 1.5 | 2.6E-04 | 104 | 668 |
| GO:0050878 | regulation of body fluid levels | 1.9 | 4.5E-04 | 39 | 197 |
| GO:0032506 | cytokinetic process | 7.9 | 8.8E-04 | 5 | 6 |
| GO:0009566 | fertilization | 2.6 | 9.6E-04 | 16 | 59 |
| GO:0065008 | regulation of biological quality | 1.6 | 1.1E-03 | 55 | 317 |
| GO:0007568 | aging | 1.8 | 1.7E-03 | 38 | 201 |
| GO:0006950 | response to stress | 1.8 | 1.8E-03 | 41 | 222 |
| GO:0008340 | multicellular organismal development: multicellular organismal aging: determination of adult life span | 1.8 | 2.8E-03 | 37 | 198 |
| GO:0010259 | multicellular organismal development: multicellular organismal aging | 1.8 | 2.8E-03 | 37 | 198 |
| GO:0006352 | transcription, DNA-dependent: transcription initiation | 3.2 | 3.8E-03 | 11 | 33 |
| GO:0046903 | secretion | 2.3 | 4.8E-03 | 17 | 71 |
| GO:0007369 | embryonic development: gastrulation | 2.2 | 4.8E-03 | 18 | 77 |
| GO:0040028 | regulation of vulval development | 2.1 | 5.6E-03 | 20 | 90 |
| GO:0009719 | response to endogenous stimulus | 1.9 | 7.7E-03 | 26 | 131 |
| GO:0006928 | cell motility | 2.0 | 8.5E-03 | 21 | 99 |
| GO:0051674 | localization of cell | 2.0 | 8.5E-03 | 21 | 99 |
| GO:0009607 | response to biotic stimulus | 3.9 | 9.4E-03 | 7 | 17 |
| GO:0009887 | multicellular organismal development: system development: organ development: organ morphogenesis | 2.4 | 1.3E-02 | 13 | 52 |
| GO:0045926 | negative regulation of growth | 2.1 | 1.5E-02 | 17 | 77 |
| GO:0019748 | secondary metabolic process | 3.5 | 1.8E-02 | 7 | 19 |
| GO:0000819 | chromosome segregation: sister chromatid segregation | 3.8 | 2.2E-02 | 6 | 15 |
| GO:0006423 | cysteinyl-tRNA aminoacylation | 7.1 | 2.9E-02 | 3 | 4 |
| GO:0045185 | maintenance of protein localization | 7.1 | 2.9E-02 | 3 | 4 |
| GO:0051093 | negative regulation of developmental process | 2.2 | 3.4E-02 | 13 | 57 |
| GO:0051128 | cellular component organization and biogenesis: regulation of cellular component organization and biogenesis | 2.9 | 4.8E-02 | 7 | 23 |
| GO:0009892 | negative regulation of metabolic process | 2.1 | 5.0E-02 | 12 | 53 |
| The 3^rd^ level GO terms from biological process are listed. The p value cutoff is 0.05. | | | | | |
