# Supplementary material for: Genome-Wide Identification of Binding Sites Defines Distinct Functions for Caenorhabditis elegans PHA-4/FOXA in Development and Environmental Response
Source: PLoS Genet. 2010 Feb 19;6(2):e1000848. doi: 10.1371/journal.pgen.1000848 (PMC2824807; doi:10.1371/journal.pgen.1000848)
Supplement: Table S3 — GO analysis of unique PHA-4 target genes in starved L1s. (0.04 MB DOC) [file pgen.1000848.s010.doc]

Table S3. GO analysis of unique PHA-4 target genes in starved L1s

| **ID** | **Name** | **Fold** | **p-value** | **Count** | **Database Count** |
| --- | --- | --- | --- | --- | --- |
| GO:0009790 | embryonic development | 0.7 | (1.5E-05) | 134 | 2752 |
| GO:0009791 | multicellular organismal development: post-embryonic development | 0.7 | (8.8E-03) | 89 | 1744 |
| GO:0007275 | multicellular organismal development | 0.8 | (1.6E-03) | 200 | 3542 |
| GO:0044238 | primary metabolic process | 1.2 | 1.9E-04 | 317 | 3555 |
| GO:0006091 | generation of precursor metabolites and energy | 1.9 | 4.0E-04 | 48 | 359 |
| GO:0006952 | defense response | 3.5 | 1.9E-03 | 13 | 51 |
| GO:0006355 | regulation of transcription, DNA-dependent | 1.5 | 8.8E-03 | 70 | 649 |
| GO:0044237 | cellular metabolic process | 1.2 | 1.4E-02 | 296 | 3495 |
| GO:0006351 | transcription, DNA-dependent | 1.4 | 3.0E-02 | 70 | 681 |
| GO:0006807 | nitrogen compound metabolic process | 1.8 | 3.5E-02 | 28 | 220 |
| GO:0045449 | regulation of transcription | 1.4 | 4.7E-02 | 73 | 729 |
| The 3^rd^ level GO terms from biological process are listed. The p value cutoff is 0.05. p value in the parenthesis represents term that is underrepresented, otherwise the term is enriched in the gene set analyzed. | | | | | |
