# Supplementary material for: Genome-Wide Identification of Binding Sites Defines Distinct Functions for Caenorhabditis elegans PHA-4/FOXA in Development and Environmental Response
Source: PLoS Genet. 2010 Feb 19;6(2):e1000848. doi: 10.1371/journal.pgen.1000848 (PMC2824807; doi:10.1371/journal.pgen.1000848)
Supplement: Table S4 — Individual functional analysis of subset of named genes. (0.06 MB DOC) [file pgen.1000848.s011.doc]

**Table S4. Individual functional analysis of subset of named genes**

|  | Shared | | Embryo-enriched | | Starved L1-enriched | |
| --- | --- | --- | --- | --- | --- | --- |
| Category | N | % | N | % | N | % |
| apoptosis/autophagy | 3 | 1.49 | 1 | 0.32 | 3 | 1.02 |
| cellular structure | 28 | 13.86 | 34 | 10.90 | 33 | 11.22 |
| chaperone | 6 | 2.97 | 12 | 3.85 | 8 | 2.72 |
| extracellular | 4 | 1.98 | 5 | 1.60 | 9 | 3.06 |
| membrane | 8 | 3.96 | 10 | 3.21 | 37 | 12.59 |
| metabolism | 21 | 10.40 | 32 | 10.26 | 71 | 24.15 |
| proteolysis | 11 | 5.45 | 28 | 8.97 | 25 | 8.50 |
| replication/repair/cell cycle | 11 | 5.45 | 15 | 4.81 | 4 | 1.36 |
| RNA metabolism | 12 | 5.94 | 18 | 5.77 | 9 | 3.06 |
| RNAi/miRNAs | 4 | 1.98 | 3 | 0.96 | 1 | 0.34 |
| signaling | 33 | 16.34 | 64 | 20.51 | 28 | 9.52 |
| sorting | 10 | 4.95 | 11 | 3.53 | 12 | 4.08 |
| transcription | 25 | 12.38 | 58 | 18.59 | 45 | 15.31 |
| translation | 26 | 12.87 | 21 | 6.73 | 9 | 3.06 |
| unknown | 8 |  | 16 |  | 17 |  |
| total | 210 |  | 328 |  | 311 |  |
| total w/o unknowns | 202 |  | 312 |  | 294 |  |

The number and percentage was calculated without including the small handful of named genes for which no known function was able to be defined. Green highlights several distinctive shifts in trends.
