# Supplementary material for: Genome-Wide Identification of Binding Sites Defines Distinct Functions for Caenorhabditis elegans PHA-4/FOXA in Development and Environmental Response
Source: PLoS Genet. 2010 Feb 19;6(2):e1000848. doi: 10.1371/journal.pgen.1000848 (PMC2824807; doi:10.1371/journal.pgen.1000848)
Supplement: Table S5 — Total number of mapped reads of ChIP-Seq experiments. (0.03 MB DOC) [file pgen.1000848.s012.doc]

**Table S5.** **Total number of mapped reads of ChIP-Seq experiments**

| **Strain** | **Stage** | **Antibody for IP** | **# mapped reads** | |
| --- | --- | --- | --- | --- |
|  |  |  | **replicate 1** | **replicate 2** |
| AMA-1 | L4 | anti-GFP | 3853569 | 6704820 |
| AMA-1 | L4 | anti-Pol II | 3169440 | 2807802 |
| AMA-1 | L4 | input | 7039397 | 2343161 |
| PHA-4 | embryo | anti-GFP | 3307483 | 4412199 |
| PHA-4 | embryo | anti-Pol II | 2100052 | 3242652 |
| PHA-4 | embryo | input | 5761117 | 4233822 |
| PHA-4 | Starved L1 | anti-GFP | 1908578 | 15936620 |
| PHA-4 | Starved L1 | anti-Pol II | 2065804 | 17097039 |
| PHA-4 | Starved L1 | input | 11316523 | 15502699 |
